# Intra-Species and Inter-Species Differences in Cytokine Production by Porcine Antigen-Presenting Cells Stimulated by *Mycoplasma hyopneumoniae*, *M. hyorhinis*, and *M. flocculare*

**DOI:** 10.3390/pathogens8010034

**Published:** 2019-03-16

**Authors:** Sarah Fourour, Corinne Marois-Créhan, Léa Martelet, Christelle Fablet, Isabelle Kempf, Marcelo Gottschalk, Mariela Segura

**Affiliations:** 1 French Agency for Food, Environmental and Occupational Health & Safety (ANSES), Ploufragan-Plouzané-Niort Laboratory, Mycoplasmology Bacteriology and Antimicrobial Resistance Unit, 22 440 Ploufragan, France; fourour.sarah@gmail.com (S.F.); corinne.marois@anses.fr (C.M.-C.); isabelle.kempf@anses.fr (I.K.); 2 University of Brittany-Loire, Cité internationale 1 place Paul Ricoeur CS 54417, 35044 Rennes, France; christelle.fablet@anses.fr; 3 Swine and Poultry Infectious Diseases Research Center and Research Group on Infectious Diseases in Production Animals, Faculty of Veterinary Medicine, University of Montreal, 3200 Sicotte St., Saint-Hyacinthe, QC J2S 2M2, Canada; lea.martelet@live.fr (L.M.); marcelo.gottschalk@umontreal.ca (M.G.); 4 French Agency for Food, Environmental and Occupational Health & Safety (ANSES), Ploufragan-Plouzané-Niort Laboratory, Epidemiology, Health and Welfare unit, 22440 Ploufragan, France

**Keywords:** porcine dendritic cells, cytokine, inflammatory response, *Mycoplasma*, pneumonia

## Abstract

*Mycoplasma hyorhinis* and *M. flocculare* are commonly co-isolated with *M. hyopneumoniae* (primary agent of swine enzootic pneumonia) in gross pneumonia-like lesions, but their involvement in the disease process remains unknown. T cells play an immuno-pathological role during mycoplasmal infections. Dendritic cells (DCs) are major antigen-presenting cells involved in T cell activation and differentiation. In this study, we investigated cytokine (IL-6, IL-8, IL-10, IL-12, and TNF-α) production by porcine bone-marrow-derived DCs (BM-DCs) stimulated by *M. hyopneumoniae*, *M. hyorhinis*, and/or *M. flocculare*. Results showed that cytokine production levels were relatively homogenous for all evaluated *M. hyopneumoniae* strains in contrast to *M. hyorhinis* and *M. flocculare* strains. The most noteworthy inter-species differences were the overall (i) lower IL-12 production capacity of *M. hyopneumoniae*, and (ii) higher TNF-α production capacity of *M. flocculare*. Co-stimulation of BM-DCs showed that *M. hyorhinis* dominated the IL-12 production independently of its association with *M. hyopneumoniae* or *M. flocculare*. In addition, a decreased BM-DC production of TNF-α was generally observed in the presence of mycoplasma associations. Lastly, *M. flocculare* association with *M. hyopneumoniae* increased BM-DC ability to secrete IL-10. A higher cytotoxicity level in BM-DCs stimulated by *M. hyorhinis* was also observed. Overall, this study demonstrated that the combination of *M. hyorhinis* or *M. flocculare* with *M. hyopneumoniae* may participate to the modulation of the immune response that might affect the final disease outcome.

## 1. Introduction

*Mycoplasma hyopneumoniae* is the causative agent of enzootic chronic pneumonia and one of the primary agents of the Porcine Respiratory Disease Complex (PRDC), which is a multifactorial disorder involving a combination of microorganisms. These diseases affecting lungs of pigs are costly for the global swine industry [1]. To control *M. hyopneumoniae* infections, commercial vaccines based on inactivated cells are commonly used worldwide [2]. Although vaccination allows the reduction of lung lesions and increases farm technical performances, it does not prevent the spread and the colonization of pig respiratory tract by this pathogen. Consequently, vaccination confers partial protection [2,3]. *M. hyopneumoniae* infection leads to the recruitment and the activation of various immune cells, essentially through the involvement of a large range of cytokines, which are secreted by several activated immune cells in response to stimuli. They have a pleiotropic role and provide crucial functions in initiation and modulation of host-defenses, including inflammatory responses. Experimentally or naturally infected pigs showed a production of interleukin (IL)-1, IL-6, and tumor necrosis factor-alpha (TNF-α) in lungs, and also IL-1, IL-2, IL-4, IL-6, IL-8, TNF-α, and IL-10 in bronchus-associated lymphoid tissue (BALT) or in tracheobronchial lavage fluid (TBLF) [4,5,6,7,8]. *In vitro* studies with murine or porcine pulmonary alveolar macrophages, important players in innate and adaptive immunity, showed IL-1α/β, IL-6, IL-8, or TNF-α involvement after an exposure to *M. hyopneumoniae* [9,10].

Despite their beneficial role, cytokines may lead to immuno-pathological events with significant host-tissues damage, especially in case of chronic or excessive production [11]. Furthermore, T cells, especially CD4^+^ T helper cells (CD4^+^ Th), are also described as a key part of immuno-pathological reactions during mycoplasma respiratory disease [12,13,14]. Because T cells play a critical role, it is essential to understand the role of antigen-presenting cells (APCs), notably dendritic cells (DCs), in T cell activation and differentiation. Cytokines produced by APCs polarize the differentiation of T cells, including Th sub-types, such as Th1 and Th2, and the subsequent development of cellular or humoral mediated responses. Generally, IL-12 is associated with Th1 responses and IL-4 or IL-6 with Th2 responses.

DCs are potent APCs with the ability to interact with T cells and polarize their differentiation into effector T cells. However, few studies have highlighted the role of DCs in the immunological response induced by mycoplasmal infections [15,16]. Coinfections with *M. hyopneumoniae* and PRDC-associated agents lead to increased inflammatory responses by the overproduction of many cytokines with consequent intensification of pulmonary tissue injures. Studies have shown changes in cytokine production when *M. hyopneumoniae* is associated with (i) Porcine Reproductive and Respiratory Syndrome virus (PRRS), which leads to increased IL-1α/β, IL-8, and TNF-α secretion by pulmonary alveolar macrophages, or (ii) Porcine circovirus (PCV)-2 leading to high levels of IFN-γ and IL-10 in tracheobronchial lymph nodes [10,17]. No study has assessed the effect of mycoplasmal association in cytokine production, even though a recent study demonstrated that *M. flocculare* or *M. hyorhinis* are co-present with *M. hyopneumoniae* in severe gross pneumonia-like lesions [18].

The aims of this study were to (i) explore cytokine production levels of porcine bone-marrow-derived DCs (BM-DCs) stimulated with different strains of *M. hyopneumoniae*, *M. hyorhinis*, or *M. flocculare*. (ii) Dissect if this production is strain and/or species-dependent, and (ii) analyze the effect of mycoplasma associations (*M. hyopneumoniae*/*M. hyorhinis* or *M. hyopneumoniae*/*M. flocculare* or *M. flocculare*/*M. hyorhinis*) on cytokine production. Results showed that mycoplasmal association may participate in the modulation of the immune response that might affect the immune defense of the host.

## 2. Results

### 2.1. Comparison of Cytokine Production Levels Induced by M. hyopneumoniae, M. hyorhinis, or M. flocculare

**Figure 1:**
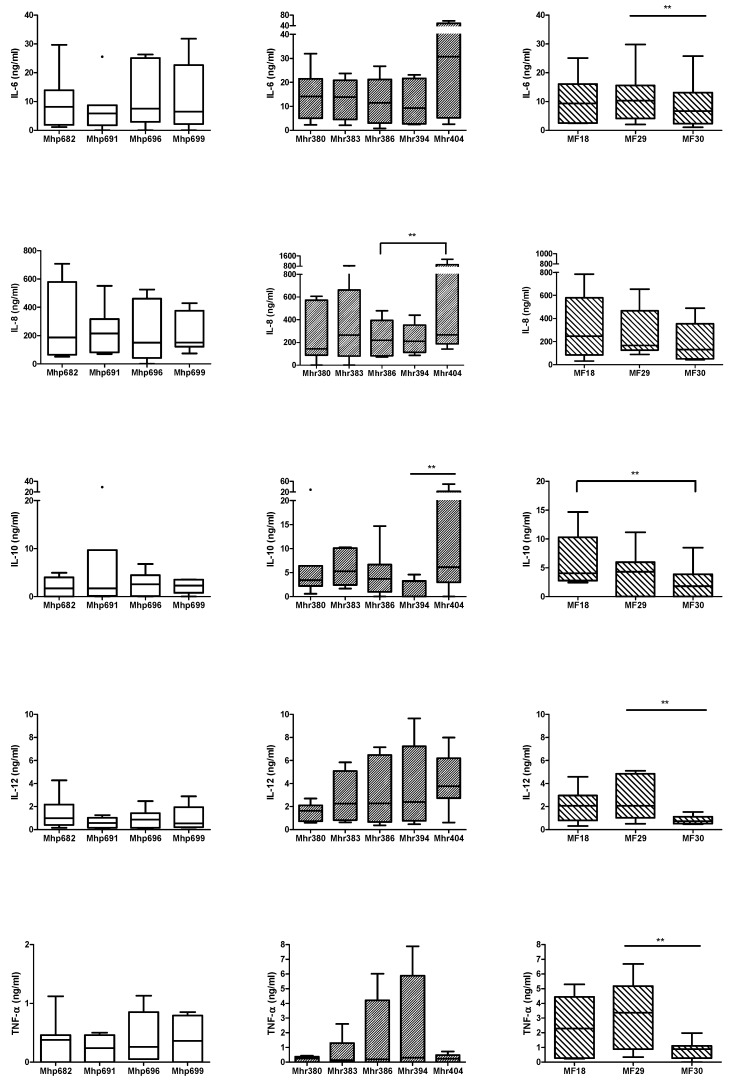
Levels of IL-6, IL-8, IL-10, IL-12, and TNF-α (expressed in ng/mL) in the culture supernatant of BM-DCs derived from seven different pigs (n = 7) and stimulated with *M. hyopneumoniae* strains (Mhp682, Mhp691, Mhp696, Mhp699), *M. hyorhinis* strains (Mhr380, Mhr383, Mhr386, Mhr394, and Mhr404) or *M. flocculare* strains (MF18, MF29, and MF30) isolated from no or mild (Mhp691, Mhr394, and MF30), moderate (Mhr383, MF18, and MF29), or severe (Mhp682, Mhp696, Mhp699, Mhr380, Mhr386, and Mhr404) gross pneumonia-like lesions, which corresponded to scores ≤2/28, 3 to 10/28, or ≥10/28, according to the notation of Madec and Kobisch [19], respectively. Data are summarized by a whisker plot including median and Interquartile Range. Values outside whiskers (outliers) are represented by dots. Values for basal cytokine production from no stimulated cells (negative control) were subtracted to cytokine concentration obtained after mycoplasmal stimulation. The mean of these values were: 1.4 ± 1.1 for IL-6, 14.3 ± 21.2 for IL-8, 0.6 ± 0.5 for IL-10, 0.4 ± 0.3 for IL-12, and 0.2 ± 0.1 for TNF-α. A *p*-value < 0.05 (*) was considered as a threshold for significance. Individual data are displayed as scatter plots in the Supplementary Figure S1.

Cytokine production by BM-DCs stimulated by all tested strains (four *M. hyopneumoniae*, five *M. hyorhinis*, and three *M. flocculare*) is presented in Figure 1 and Figure S1. All strains were able to produce IL-6, IL-8, IL-10, IL-12, and TNF-α, and variable levels were observed. Concerning *M. hyopneumoniae* strains, no intra-species significant difference was found in the production of all tested cytokines. On the contrary, *M. hyorhinis* and *M. flocculare* strains appeared to be more heterogeneous. However, *M. hyorhinis* strains induced the same production levels of IL-6, IL-12, and TNF-α. Statistically significant differences were found in the production of IL-8 and IL-10 for some strains. The Mhr404 strain produced higher concentration of these two cytokines, compared to Mhr386 (*p* = 0.03) and Mhr394 (*p* = 0.03), respectively. *M. flocculare* strains were the most variable with inter-strain cytokine production level differences observed for all evaluated cytokines, except IL-8.

When data of cytokine concentration were analyzed by species, the average production levels of IL-10 and IL-12 were statistically higher for *M. hyorhinis* when compared to *M. hyopneumoniae* (*p* = 0.03 and *p* < 0.01, respectively). Moreover, a trend toward a higher average concentration of IL-12 was also observed for *M. flocculare* compared to *M. hyopneumoniae* (*p* = 0.09). Consequently, *M. hyopneumoniae* induced an overall lower production level of IL-12 when compared to other mycoplasmas. The average production level of TNF-α was statistically higher for *M. flocculare* when compared to *M. hyopneumoniae* or *M. hyorhinis* (both *p* < 0.01). Overall, *M. hyopneumoniae* seems to possess the lowest pro-inflammatory stimulating capacity.

### 2.2. Comparison of Cytokine Production Levels between M. hyopneumoniae, M. hyorhinis, and M. flocculare, Alone or in Association

#### 2.2.1. Association between *M. flocculare* and *M. hyopneumoniae* Strains

**Figure 2:**
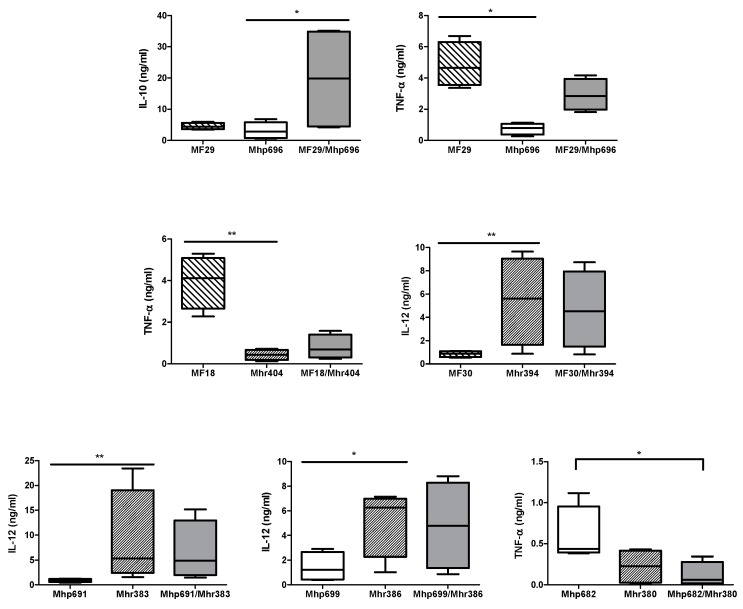
Levels of IL-10, IL-12, and TNF-α (expressed in ng/mL) produced by BM-DCs derived from four different pigs (n = 4) and stimulated with *M. hyopneumoniae* (Mhp) and/or *M. flocculare* (MF) and/or *M. hyorhinis* (Mhr) strains isolated from no or mild (Mhp691, Mhr394, MF30), moderate (Mhr383, MF18, MF29), or severe (Mhp696, Mhp699, Mhr386, Mhr404) gross pneumonia-like lesions, which corresponded to scores ≤2/28, 3 to 10/28, or ≥10/28, according to the notation of Madec and Kobisch [19], respectively. Combined strains were isolated from two different pigs of the same herd. Values for basal cytokine production from no stimulated cells (negative control) were subtracted to cytokine concentration obtained after mycoplasmal stimulation. Data are summarized by a whisker plot including a median and an Interquartile Range. The mean of these values were: 0.5 ± 0.4 for IL-10, 0.5 ± 0.4 for IL-12, and 0.2 ± 0.1 for TNF-α. A *p*-value < 0.05 (*) was considered as a threshold for significance.

BM-DCs stimulated by association of *M. flocculare* and *M. hyopneumoniae* strains (combination named MF29/Mhp696) revealed a trend toward a higher production level of IL-10 when compared to the same BM-DCs stimulated by Mhp696 alone (*p* = 0.09) (Figure 2), but do not produce a significant higher level compared to MF29 alone (*p* = 0.54). Therefore, these results failed to determine a synergic effect between strains MF29 and Mhp696, but it was clearly noted that co-stimulation does appear to produce higher levels of IL-10 when compared to Mhp696 alone.

BM-DCs stimulated by strain MF29 alone produced a significantly higher concentration of TNF-α compared to BM-DCs stimulated by Mhp696 alone (*p* = 0.01). TNF-α levels induced by the combined strains MF29/Mhp696, however, were low and similar to Mhp696 alone, which suggests that Mhp696 could reduce the ability of MF29 to induce TNF-α production (Figure 2).

No significant effects of this association on other tested cytokines were observed versus the individual strains (Supplementary Figure S2). Thus, IL-10 seems to be particularly targeted when *M. flocculare* and *M. hyopneumoniae* are found together.

#### 2.2.2. Association between *M. flocculare* and *M. hyorhinis* Strains

Among all cytokines tested, particular findings were remarked in the ability of BM-DCs to produce IL-12 and TNF-α when stimulated by the association of *M. hyorhinis* and *M. flocculare* (two combinations tested, named MF18/Mhr404 and MF30/Mhr394) (Figure 2). BM-DCs stimulated by MF18 alone produced a significantly higher average concentration of TNF-α when compared to BM-DCs stimulated by Mhr404 alone (*p* = 0.04). In spite of no statistically significant difference among individual strains and the association, a reduction of MF18 strain ability to induce TNF-α production in the presence of Mhr404 was observed. On the other hand, BM-DCs stimulated by Mhr394 were able to produce a higher concentration of IL-12 when compared to BM-DCs stimulated by MF30 (*p* = 0.05). However, the ability of Mhr394 to induce this high production of IL-12 stayed unchanged in the presence of MF30. Consequently, strain Mhr394 appears to dominate this production with neither additive nor a synergistic effect that was observed.

#### 2.2.3. Association between *M. hyorhinis* and *M. hyopneumoniae* Strains

Once again, among all cytokines tested, we focused our attention on the ability of BM-DCs to produce IL-12 and TNF-α when stimulated by the association of *M. hyopneumoniae* and *M. hyorhinis*. Three combinations, named Mhp682/Mhr380, Mhp691/Mhr383 and Mhp699/Mhr386, were evaluated (Figure 2). Mhr383 and Mhr386 strains appear to dominate the IL-12 production compared to *M. hyopneumoniae* strains, Mhp691 and Mhp699 (*p* = 0.04 and *p* = 0.09, respectively). The ability of these two *M. hyorhinis* strains to induce the production of IL-12 stayed unchanged in the presence of *M. hyopneumoniae* strains, Mhp691 and Mhp699 (*P* > 0.05). Therefore, as mentioned previously, *M. hyorhinis* strains appear to dominate the IL-12 production with neither an additive nor a synergistic effect observed in the presence of other mycoplasmas. BM-DCs stimulated by Mhp682 and Mhr380 strains alone were able to produce the same levels of TNF-α. However, a significantly lower concentration was observed for the combined strains Mhp682/Mhr380 when compared to Mhp682 alone (*p* = 0.04). Therefore, Mhr380 decreased the ability of Mhp682 to induce the production of TNF-α.

### 2.3. Viability of BM-DCs Stimulated by M. hyopneumoniae, M. hyorhinis, and M. flocculare Species

**Figure 3:**
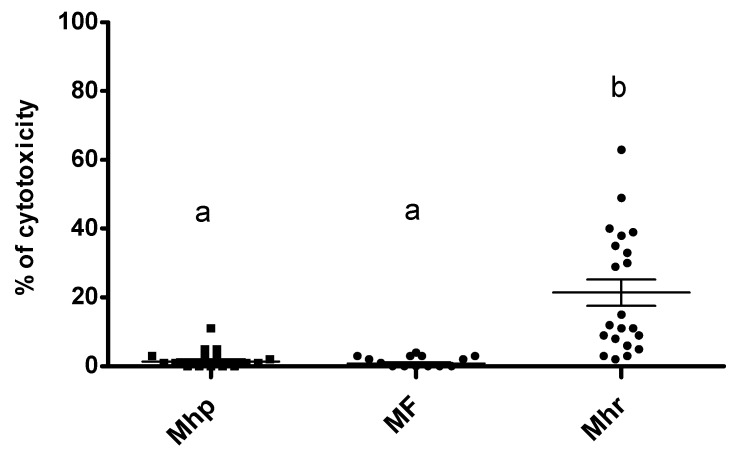
Cell toxicity induced by *M. hyopneumoniae* (Mhp), *M. hyorhinis* (Mhr), or *M. flocculare* (MF) strains assessed by measuring the lactate dehydrogenase (LDH) released in the supernatant of stimulated cells. Values are grouped by species and include three repetitions performed with BM-DCs derived from three different pigs. Data are represented by dots and the mean is symbolized by a bold line ± SEM. A *p*-value < 0.05 was considered as a threshold for significance. Different letters (a, b) indicate significant differences.

Results grouped by species showed no cytotoxicity effect for *M. hyopneumoniae* and *M. flocculare* strains on stimulated BM-DCs. However, a significantly higher percentage of cytotoxicity was measured for *M. hyorhinis* strains (*p* < 0.001) (Figure 3). For mycoplasma combinations, no differences were detected when *M. hyorhinis* was alone when compared to when it was in combination with other mycoplasma species (data not shown). Thus, the cytotoxic effect was intrinsic to *M. hyorhinis*.

## 3. Discussion

The purpose of this study was to assess the level of IL-6, IL-8, IL-10, IL-12, and TNF-α produced by porcine BM-DCs stimulated by contemporary strains of *M. hyopneumoniae*, *M. hyorhinis*, and *M. flocculare*, alone or in association. Because the genome of mycoplasma evolution vary quickly and the virulence is often described as strain-dependent, we included several strains isolated in 2016 in this study from the same or different herds and from lungs with different extensions of gross pneumonia-like lesions obtained in a previous study [18]. The selected BM-DC model was already applied to predict the potential induction of local inflammatory responses or the Th1/Th2 pathways by swine pathogens such as *Streptococcus suis* or *M. hyopneumoniae* [15,20,21,22]. Most studies have assessed cytokine production in lungs, BALT, or TBLF of infected pigs [4,5,6,7,8]. However, DC ability to produce a large range of cytokines could also be involved in immuno-pathogenicity during mycoplasmal respiratory infections [16]. A single recent study has investigated the effect of *M. hyopneumoniae* in DC functions, and results showed a decline of their antigen presentation ability, which is possibly responsible of infection persistence [15]. The originality of our *in vitro* study, therefore, lies in the investigation of production of a large number of cytokines (IL-6, IL-8, IL-10, IL-12, and TNF-α) by porcine BM-DCs after stimulation with 12 contemporary strains from three mycoplasmal species.

Since mycoplasmas lack a cell wall, mechanisms of interaction with host cells are poorly understood. Lipoproteins are described as playing a key role at the interface between mycoplasmas and immune cells [23,24]. Our results showed that all evaluated strains initiate the production of all tested cytokines, which indicates an interaction between mycoplasmal components and BM-DCs receptors. This type of interaction has never been described for *M. flocculare*, which is considered as being, according to different authors, either a commensal of the respiratory tract or a potential PRDC-associated agent [1,25].

The other aim of our study was to investigate the intra-species differences in cytokine production levels. Evaluated *M. hyopneumoniae* strains appear to be overall homogeneous while Woolley et al. [26] showed differences in the ability of two *M. hyopneumoniae* strains to induce cytokine production. The authors correlated it with the development of clinical signs and tissue lesions. In contrast, *M. hyorhinis* and *M. flocculare* strains seem to be more heterogeneous since differences in production levels of several tested cytokines were found among strains of these species. One strain of *M. hyorhinis* (Mhr394) and one of *M. flocculare* (MF30) appear to be peculiar since they induced the lowest levels of some cytokines. MF30 and Mhr394 were isolated from the same herd with a low average batch level score of gross pneumonia-like lesions (1.4/28 from a sample of fifteen lungs, scoring described by Madec and Kobisch [19]). Similarly, strains used for some of the mycoplasmal species associations, and resulting in higher levels of some cytokine production than single infections, came from two herds having a high average score of gross pneumonia-like lesions (>7/28).

In addition to intra-species differences (strain-specific responses), inter-species differences were observed. The most notable were: (i) the overall lower IL-12 production capacity of *M. hyopneumoniae* unlike to *M. hyorhinis* and (ii) the overall higher TNF-α production capacity of *M. flocculare* compared to other mycoplasmal species. IL-12 acts as an antagonist of Th2 humoral pathway by the inhibition of IL-4 production. Therefore, it indirectly favors the Th1 pathway. Our results, thus, suggest a preferentially Type 2 response of BM-DCs exposed to *M. hyopneumoniae*, which is in agreement with another study [15]. The Th2 response is reported as non-efficient against mycoplasma infections, which could explain the persistence of the infection [15]. TNF-α is an important pro-inflammatory cytokine involved in the secretion of acute-phase proteins and is frequently detected in *M. hyopneumoniae*-infected pigs [4,5,6,7,8]. Furthermore, a high TNF-α concentration could be responsible for growth retardation and tissue inflammatory reactions [27]. In contrast to *M. flocculare*, a low TNF-α production level was observed for *M. hyopneumoniae*. This is the first study that demonstrates that *M. flocculare* could play an initial role in pulmonary inflammation by inducing the production of TNF-α by BM-DCs. However, more investigations are necessary to fully elucidate the role of TNF-α in the development of pneumonia because its real implication is not completely understood [28]. Additional studies with a higher number of strains per species will be required to better dissect the inter-species differences in cytokine production.

Another recent study has shown that mycoplasma associations were essentially found in lungs with extensive gross pneumonia-like lesions [18]. This result led us to investigate potential changes in cytokine production when BM-DCs are co-stimulated by two mycoplasma species. One unique finding is the increased IL-10 production by BM-DCs stimulated by *M. hyopneumoniae* in combination with *M. flocculare*. According to the local concentration, IL-10 can act as an anti-inflammatory cytokine necessary to maintain cell homeostasis or favor Th2 pathway by inhibiting IL-12 production [29]. Increased IL-10 levels were also described in the case of an association between *M. hyopneumoniae* and PRRS virus and could be responsible for the duration of infections by modulating the immune response [30]. Since only one combination of *M. flocculare* and *M. hyopneumoniae* strains was used, further works with a higher number of strains should be done to assess if this is a common pattern observed when these two species are found together.

Overall, *M. hyorhinis* strains are strong inducers of IL-12. The association with *M. hyopneumoniae* or *M. flocculare* does not influence this capacity. A competitive binding for BM-DCs receptors in favor of *M. hyorhinis* may explain these results or this intrinsic stimulatory activity exceeds activation signals induced by other mycoplasmas. It remains to be elucidated if the IL-12 favored by *M. hyorhinis* has a beneficial or pathological effect in the presence of *M. flocculare* or *M. hyopneumoniae* during natural infections. The strong capacity of *M. hyorhinis* to induce cytokine production (mainly IL-12) by BM-DCs was observed in spite of a moderate cytotoxicity induced under cultural conditions. This study does not allow us to conclude if the lysis of cells is a defense mechanism used by the host to reduce the internalization of *M. hyorhinis* or if BM-DCs were lysed by the production of cytotoxic components by this mycoplasma. Contrary to our results with porcine BM-DCs, *M. hyopneumoniae* was reported to induce cytotoxicity in macrophages or peripheral blood mononuclear cells [31]. A diminution of host immune cells, by mortality or by inhibition of proliferation, could lead to an impairment of the implantation of the immune response and pathogen persistence. Lastly, a trend to a reduced production of TNF-α when two mycoplasma species are found together was observed, which suggests that the role of this cytokine in the pathogenesis of the disease is multi-faceted and partially clarified.

In conclusion, complex patterns of BM-DC activation by mycoplasma were observed, which differ at the intra-species and inter-species levels and were modulated by the presence of mycoplasma associations. Since a single cell-type culture model does not completely represent the complexity of cell interactions, future *in vivo* studies will allow unravelling of the implications of these findings in the pathogenesis of mycoplasma infections.

## 4. Materials and Methods

### 4.1. Mycoplasma Strains and Growth Conditions

A total of 12 field strains isolated from lungs with gross pneumonia-like lesions, from pigs reared in the same or different herds with variable levels of respiratory disease were used (Table 1). All mycoplasma strains were cultured in Friis liquid medium as described [32] at 37 °C, until the beginning of the stationary phase observed by the shift of the red color medium to the orange color due to the phenol red pH indicator. Then, cultures were centrifuged to collect the pellet (12,000 *g*, 15 min, 4 °C), which was washed twice with phosphate-buffered saline (PBS) solution and suspended with 1 mL of complete Roswell Park Memorial Institute tissue culture medium 1640 (RPMI) supplemented with 10% heat-inactivated fetal bovine serum (FBS), 2 mM L-glutamine, 10 mM HEPES, 100 IU/mL penicillin-streptomycin, and 1 µg/mL gentamicin (Invitrogen, Burlington, ON, Canada). All solutions and materials were endotoxin-free.

### 4.2. Isolation of Bone-Marrow Cells and Generation of Bone-Marrow-Derived Dendritic Cells (BM-DCs)

Seven pigs were housed under specific pathogen-free conditions and euthanized at six weeks old. The animals originated from a herd free of major important diseases such as PRRS, enzootic pneumonia due to *M. hyopneumoniae*, and clinical disease related to porcine circovirus. All experiments involving animals were conducted in accordance with the guidelines and policies of the Canadian Council on Animal Care and the principles set forth in the Guide for the Care and Use of Laboratory Animals and approved by the Animal Welfare Committee of the University of Montreal (protocol Rech-1570). Femurs of each animal were aseptically extracted in animal facilities and were transported on ice to the laboratory. The extraction and the culture of bone-marrow cells were performed as described by Martelet et al. [21]. The muscle tissue was completely removed and bones were sliced in one centimeter and stirred in 1 L of PBS for 2 h at room temperature. After a filtration through gauzes, the suspension was centrifuged (250 *g*, 10 min, 4 °C) and the obtained pellet was treated with red blood cell lysis reagent (eBioScience, San Diego, CA, USA). Cells were washed and filtered through a 40 μm cell strainer (BD FalconTM, Bedford, MA, USA). The obtained bone-marrow cells were suspended (at approximately 10^7^ cells/mL) in a cryopreservation solution containing 95% of FBS and 5% of dimethylsulfoxyde (DMSO) (Sigma-Aldrich, Oakville, ON, Canada). Bone-marrow cells were stored in liquid nitrogen until use.

The conserved bone-marrow cells were suspended and washed in complete RPMI medium, and then centrifuged (250 *g*, 10 min, 4 °C) to recover the pellet. Cells were counted with the TC20TM automated cell counter (Biorad, Hercules, CA, USA), and the concentration was adjusted to 10^6^ cells/mL and distributed in six-well culture plates (Falcon^®^, Corning, Tewksbury, MA, USA). Porcine Granulocyte-Macrophage Colony Stimulating Factor (GM-CSF), produced in house from a CHO-K1/pGM-CSF stable cell line as described by Martelet et al. [21], was added at 1/50 dilution to each well. The cells were incubated at 37 °C and 5% of CO_2_. At day three and six, medium was replaced by fresh complete RPMI medium to remove non-adherent cells. On day 8, bone-marrow cells had acquired an immature BM-DCs phenotype by microscopic observation, which was confirmed by FACS as MHC-I^+^, MHC-II^+^, SWC3^+^, CD1^+^, CD16^+^, CD14^+^, CD11R1^−^, and CD4a^low/−^, as previously described [21]. This culture system cannot completely rule out the presence of other innate cells (such as macrophages), it thus represents an enriched source of BM-DCs.

### 4.3. Stimulation of Immature BM-DCs by Mycoplasma Strains

At day 8, immature BM-DCs were collected and counted, and cells were distributed in 24-well culture plate (Falcon^®^) at a concentration of 2 × 10^6^ cells/well in a total volume of 500 µL. For mono-stimulation (stimulation of BM-DCs by a single strain), 500 µL of mycoplasma suspension containing ~10^8^ mycoplasmal cells was added to wells containing BM-DCs. A total of 12 strains were tested in seven repetitions corresponding to BM-DCs derived from seven different pigs (n = 7). For co-stimulation (stimulation of BM-DCs by two strains belonging to two different species), each mycoplasma suspension was concentrated (2×) to have the same amount of each mycoplasma strains as in the mono-stimulation. The combination between species was performed to associate strains isolated from the same herd. A total of six combinations were tested (Mhp682/Mhr380, Mhp691/Mhr383, Mhp699/Mhr386, Mhp696/MF29, MF18/Mhr404, and MF30/Mhr394) in four repetitions corresponding to BM-DCs derived from four different pigs (n = 4). Negative and positive controls were performed with either complete RPMI medium alone or 2 µg/mL of lipopolysaccharide (LPS) (from *Escherichia coli* 0127:B8, Sigma-Aldrich), respectively, which was directly added to wells containing BM-DCs. Control cells or BM-DCs with mycoplasma strains were incubated for 24 h at 37 °C. In addition, 5% CO_2_ and supernatants were collected for cytokine quantification.

### 4.4. Cytokine Quantification by Enzyme Linked Immunosorbent Assay (ELISA)

Levels of IL-12, IL-6, IL-8, IL-10, and TNF-α in cell culture supernatants were measured by sandwich ELISA using pair-matched antibodies from R&D Systems (Minneapolis) for TNF-α, IL-6, and IL-8. An R&D Systems duoset kit was used for IL-12. Pair-matched antibodies from Invitrogen (Burlington, ON, Canada) were used for IL-10, according to the manufacturer’s recommendations. Twofold dilutions of recombinant porcine cytokines were used to generate the standard curves. Sample dilutions giving optical density readings in the linear portion of the appropriate standard curve were used to quantify the levels of each cytokine. For each cytokine, the value of the negative control was subtracted.

### 4.5. Cell Viability Assay

Viability of BM-DCs during the incubation period with mycoplasma strains was tested by measuring the lactate dehydrogenase (LDH) released in the supernatant of BM-DCs derived from three different pigs (n = 3). The quantification of LDH was performed using the CytoTox 96 Non-Radioactive Cytotoxicity assay kit (Promega, Madison, WI, USA), according to the manufacturer’s instructions.

### 4.6. Statistical Analysis

#### 4.6.1. Strain and Species Effects of *M. hyopneumoniae*, *M. hyorhinis,* or *M. flocculare* on Cytokine Production Levels

The aim is to study if the cytokine production level depends on (i) the strains (comparison of cytokine production between all strains within the same species), or (ii) the species (comparison of cytokine production between pool of strains by species) (i.e., average production by species). The relationships between *M. hyopneumoniae*, *M. hyorhinis*, or *M. flocculare* strains and cytokine production levels were assessed using a Friedman paired test (*p* < 0.05), followed by the Nemenyi’s post-hoc test when significant difference was found. The relationships between *M. hyopneumoniae*, *M. hyorhinis*, and *M. flocculare* species and the IL-8, IL-10, IL-12, and TNF-α cytokine production level were analyzed for significance using a Kruskall-Wallis unpaired test following by a Kolmogorov-Smirnov test to compare the groups in pairs when a difference was found. For IL-6, since data followed a normal distribution with homogeneity of variance, they were tested for significance using ANOVA following Tukey’s post-hoc test when a significant difference was found. A *p*-value < 0.05 was considered for significance and a *p*-value < 0.1 was considered for a trend. Statistical tests were performed using R software (R development Core Team 2008) and SYSTAT software.

#### 4.6.2. Mycoplasmal Co-Infection Effect on Cytokine Production Levels

The aim is to determine if the cytokine production level changes when BM-DCs are stimulated by one strain when compared to the same BM-DCs co-stimulated by two strains of different species. The methodology used is the same as mentioned above, with the exception that cytokine concentrations were assessed with BM-DCs derived from four different pigs (n = 4). Analysis for significance were performed using a Friedman paired and non-parametric test, following by the Nemenyi’s post-hoc test when a difference was found. A *p*-value < 0.05 was considered for significance and a *p*-value < 0.1 was considered for a trend.

#### 4.6.3. Cytotoxicity effect of *M. hyopneumoniae*, *M. hyorhinis*, and *M. flocculare* on BM-DCs

The cytotoxicity effect of mycoplasmal strains on BM-DCs was studied with BM-DCs derived from three different pigs (n = 3). Data were grouped by species and the cytotoxicity level was compared using a Kruskal-Wallis non-parametric test, which was followed by a Kolmogorov-Smirnov test to compare the groups in pairs. Statistical significance is defined by a *p*-value < 0.05. Analyses were performed using SYSTAT software.

## Figures and Tables

**Table 1:** Description of the field *Mycoplasma* spp. strains used in the study ^1^.

Mycoplasma Strains	Herds	Gross Pneumonia-Like Lesion Score	
		Lung Score where the Strain Was Isolated (/28)	Average Score of 15 Lungs from the Herd (/28)
*Mycoplasma hyopneumoniae* 682 (Mhp682)	a	17	10.8
*Mycoplasma hyopneumoniae* 691 (Mhp691)	b	0	10.4
*Mycoplasma hyopneumoniae* 696 (Mhp696)	c	15	7.3
*Mycoplasma hyopneumoniae* 699 (Mhp699)	d	11	10.9
*Mycoplasma hyorhinis* 380 (Mhr380)	a	17	10.8
*Mycoplasma hyorhinis* 383 (Mhr383)	b	5	10.4
*Mycoplasma hyorhinis* 386 (Mhr386)	d	12	10.9
*Mycoplasma hyorhinis* 394 (Mhr394)	f	2	1.4
*Mycoplasma hyorhinis* 404 (Mhr404)	e	19	8.9
*Mycoplasma flocculare* 18 (MF18)	e	6	8.9
*Mycoplasma flocculare* 29 (MF29)	c	8	7.3
*Mycoplasma flocculare* 30 (MF30)	f	0	1.4

^1^ All strains were isolated in 2016 from lungs of different pigs from herds with respiratory disorders. Each lung was scored for gross pneumonia-like lesions, according to the method described by Madec and Kobisch [19]. The average score was calculated from a sample of 15 lungs of pigs within the selected herd. A score of “0” indicates a healthy animal. Letters (“a” to “f”) were attributed to strains, according to the herds from which they were isolated.

## Supplementary Materials

The following are available online at https://www.mdpi.com/2076-0817/8/1/34/s1, Figure S1: Levels of IL-6, IL-8, IL-10, IL-12 and TNF-α in the culture supernatant of BM-DCs stimulated with *M. hyopneumoniae*, *M. hyorhinis* or *M. flocculare* strains, Figure S2: Levels of IL-6, IL-8 and IL-12 in the culture supernatant of BM-DCs stimulated with *M. hyopneumoniae* or *M. flocculare* strains alone or in combination.
