# Supplementary material for: Intra-Species and Inter-Species Differences in Cytokine Production by Porcine Antigen-Presenting Cells Stimulated by Mycoplasma hyopneumoniae, M. hyorhinis, and M. flocculare
Source: Pathogens. 2019 Mar 16;8(1):34. doi: 10.3390/pathogens8010034 (PMC6471550; doi:10.3390/pathogens8010034)
Supplement: Supplementary file 1 [file pathogens-08-00034-s001.pdf]

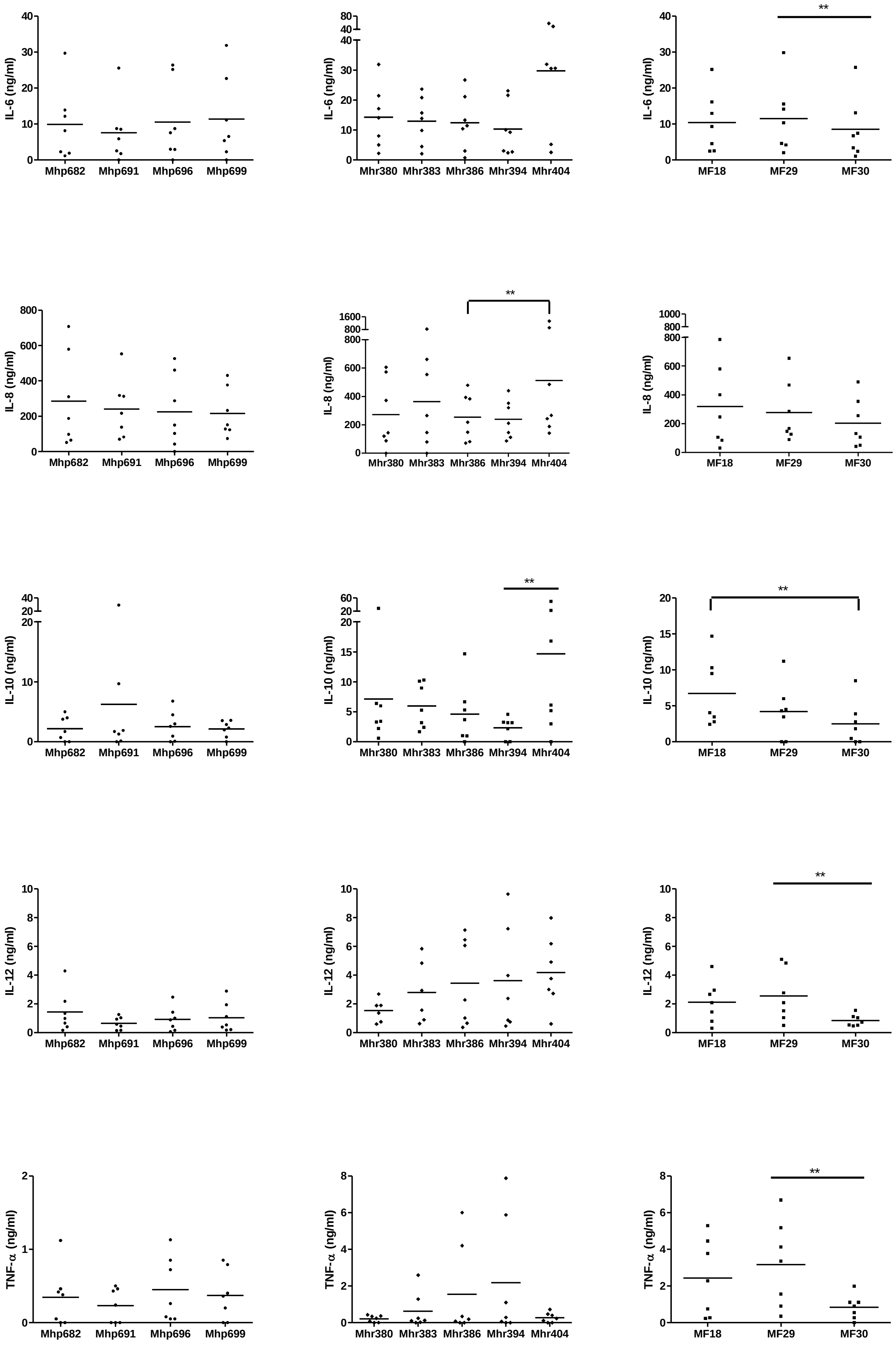

IL-6 (ng/ml)
0 10 20 30 40
Mhp682 Mhp691 Mhp696 Mhp699
IL-6 (ng/ml)
0 10 20 30 40 40 80
Mhr380 Mhr383 Mhr386 Mhr394 Mhr404
**
IL-6 (ng/ml)
0 10 20 30 40
MF18 MF29 MF30
IL-8 (ng/ml)
0 200 400 600 800
Mhp682 Mhp691 Mhp696 Mhp699
**
IL-8 (ng/ml)
0 200 400 600 800 800 1600
Mhr380 Mhr383 Mhr386 Mhr394 Mhr404
IL-8 (ng/ml)
0 200 400 600 800 800 1000
MF18 MF29 MF30
IL-10 (ng/ml)
0 10 20 20 40
Mhp682 Mhp691 Mhp696 Mhp699
**
IL-10 (ng/ml)
0 5 10 15 20 20 60
Mhr380 Mhr383 Mhr386 Mhr394 Mhr404
**
IL-10 (ng/ml)
0 5 10 15 20
MF18 MF29 MF30
IL-12 (ng/ml)
0 2 4 6 8 10
Mhp682 Mhp691 Mhp696 Mhp699
IL-12 (ng/ml)
0 2 4 6 8 10
Mhr380 Mhr383 Mhr386 Mhr394 Mhr404
**
IL-12 (ng/ml)
0 2 4 6 8 10
MF18 MF29 MF30
TNF-α (ng/ml)
0 1 2
Mhp682 Mhp691 Mhp696 Mhp699
TNF-α (ng/ml)
0 2 4 6 8
Mhr380 Mhr383 Mhr386 Mhr394 Mhr404
**
TNF-α (ng/ml)
0 2 4 6 8
MF18 MF29 MF30

Supplementary Figure S1. Levels of IL-6, IL-8, IL-10, IL-12 and TNF-α (expressed in ng/ml) in the culture supernatant of BM-DCs derived from seven different pigs (n = 7) and stimulated with *M. hyopneumoniae* strains (Mhp682, Mhp691, Mhp696, Mhp699), *M. hyorhinis* strains (Mhr380, Mhr383, Mhr386, Mhr394, Mhr404) or *M. flocculare* strains (MF18, MF29, MF30) isolated from no or mild (Mhp691, Mhr394, MF30), moderate (Mhr383, MF18, MF29) or severe (Mhp682, Mhp696, Mhp699, Mhr380, Mhr386, Mhr404) gross pneumonia-like lesions, which corresponded to scores ≤2/28, 3 to 10/28 or ≥10/28 according to the notation of Madec and Kobisch [19], respectively. Data are summarized by a scatter plot including mean ± standard error of the mean. Values for basal cytokine production from no stimulated cells (negative control) were subtracted to cytokine concentration obtained after mycoplasmal stimulation. The mean of these values were: 1.4 ± 1.1 for IL-6; 14.3 ± 21.2 for IL-8; 0.6 ± 0.5 for IL-10; 0.4 ± 0.3 for IL-12; 0.2 ± 0.1 for TNF-α. A P-value < 0.05 (*) was considered as a threshold for significance.

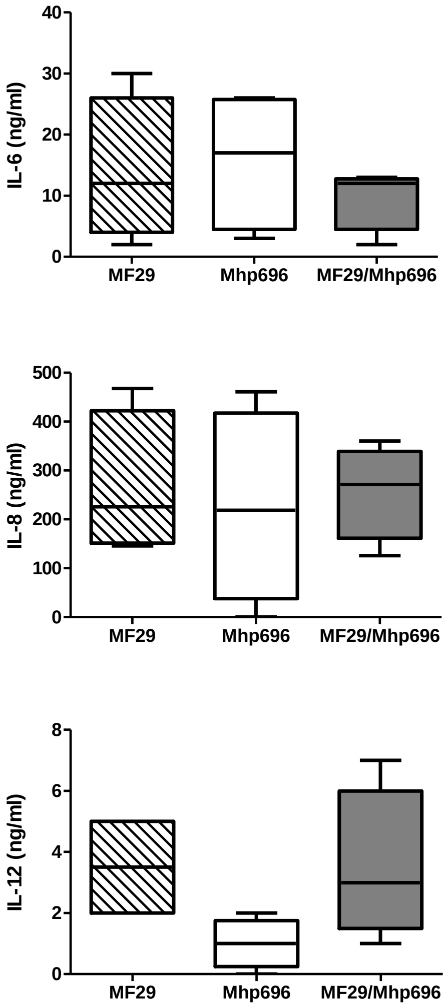

Supplementary Figure S2. Levels of IL-6, IL-8 and IL-12 (expressed in ng/ml) in the culture supernatant of BM-DCs derived from four different pigs (n = 4) and stimulated with *M. hyopneumoniae* strain 696 (Mhp696) or *M. flocculare* strain (MF29) alone or in combination. Mhp696 and MF29 were isolated from two pigs (of the same herd) presenting severe or moderate pneumonia-like lesions which corresponded to scores ≥10/28 or 3 to 10/28 according to the notation of Madec and Kobisch [19], respectively. Data are summarized by a whisker plot including median and Interquartile Range. Values for basal cytokine production from no stimulated cells (negative control) were subtracted to cytokine concentration obtained after mycoplasmal stimulation. The mean of these values were: 1.0 ± 0.7 for IL-6; 17.3 ± 27.7 for IL-8; and 0.5 ± 0.4 for IL-12.
